# A Modular Fluorescent Probe for Viscosity and Polarity Sensing in DNA Hybrid Mesostructures

**DOI:** 10.1002/advs.202003740

**Published:** 2020-12-23

**Authors:** Simon Ludwanowski, Avik Samanta, Sebastian Loescher, Christopher Barner‐Kowollik, Andreas Walther

**Affiliations:** ^1^ Institute for Macromolecular Chemistry University of Freiburg Stefan‐Meier‐Straße 31 Freiburg 79104 Germany; ^2^ Freiburg Materials Research Center (FMF) University of Freiburg Stefan‐Meier‐Straße 21 Freiburg 79104 Germany; ^3^ Freiburg Center for Interactive Materials and Bioinspired Technologies (FIT) University of Freiburg Georges‐Köhler‐Allee 105 Freiburg 79110 Germany; ^4^ Centre for Material Science School of Chemistry Physics and Mechanical Engineering Queensland University of Technology (QUT) 2 George Street Brisbane QLD 4000 Australia; ^5^ Macromolecular Architectures Institute for Technical Chemistry and Polymer Chemistry Karlsruhe Institute of Technology (KIT) Engesserstr. 18 Karlsruhe 76128 Germany; ^6^ Cluster of Excellence livMatS @ FIT – Freiburg Center for Interactive Materials and Bioinspired Technologies University of Freiburg Georges‐Köhler‐Allee 105 Freiburg D‐79110 Germany

**Keywords:** DNA mesostructures, molecular probes, protocells, Stokes shift, twisted intramolecular charge transfer, viscosity probes

## Abstract

There exists a critical need in biomedical molecular imaging and diagnostics for molecular sensors that report on slight changes to their local microenvironment with high spatial fidelity. Herein, a modular fluorescent probe, termed StyPy, is rationally designed which features i) an enormous and tunable Stokes shift based on twisted intramolecular charge transfer (TICT) processes with no overlap, a broad emission in the far‐red/near‐infrared (NIR) region of light and extraordinary quantum yields of fluorescence, ii) a modular applicability via facile *para*‐fluoro‐thiol reaction (PFTR), and iii) a polarity‐ and viscosity‐dependent emission. This renders StyPy as a particularly promising molecular sensor. Based on the thorough characterization on the molecular level, StyPy reports on the viscosity change in all‐DNA microspheres and indicates the hydrophilic and hydrophobic compartments of hybrid DNA‐based mesostructures consisting of latex beads embedded in DNA microspheres. Moreover, the enormous Stokes shift of StyPy enables one to detect multiple fluorophores, while using only a single laser line for excitation in DNA protocells. The authors anticipate that the presented results for multiplexing information are of direct importance for advanced imaging in complex soft matter and biological systems.

## Graphical abstract

The rational design of StyPy enables a push–pull system that undergoes a twisted intramolecular charge transfer (TICT) in its excited state and is applied to sense local viscosities and polarities in DNA hybrid mesostructures. The enormous Stokes shift of 250 nm enables to concurrently detect StyPy and conventional dyes with a single laser excitation.

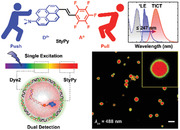

## 1 Introduction

The bottom‐up molecular engineering of chromophores with a large bathochromic shift into the far‐red/near‐infrared (NIR) region (>650 nm) is essential to their applications in the field of biomedical imaging and therapeutics.^[^ ^1^ ^]^ NIR light has a high tissue penetrating aptitude (>5 mm) via experiencing a bare minimum absorption by the major constituents of blood, namely oxy‐ and deoxy‐hemoglobin as well as water, while diminishing autofluorescence and scattering.^[^ ^2^ ^]^ There are numerous approaches to obtain a large bathochromic shift in a chromophore, for example, by extending the core conjugation (e.g., cyanine dyes)^[^ ^3^ ^]^ or by introducing a pair of electron‐donating (EDG) and electron‐withdrawing (EWG) groups, enabling a push–pull system.^[^ ^4^ ^]^ Even though chemists have been employing these molecular design principles since the beginning of the 20th century—the azo scaffolds being the eminent contender^[^ ^5^ ^]^—significant advances are possible towards multimodal imaging probes, when the chromophore design allows simultaneous reporting of even slight changes of its local microenvironment (e.g., polarity, viscosity, etc.).^[^ ^6^ ^]^ For example, Ogilby and coworkers used a porphyrin dimer to photo‐induce cell death caused by singlet oxygen, while reporting the intracellular viscosity.^[^ ^7^ ^]^ Although there has been substantial progress in the field of fluorescent molecular sensors,^[^ ^8^ ^]^ the employed chromophores generally require a complex synthesis while fulfilling only one specific task.

In 1973, Grabowski and coworkers proposed that fluorophores equipped with a pair of strong EDG and EWG can undergo a twisted intramolecular charge transfer (TICT) upon excitation.^[^ ^9^ ^]^ The donor/acceptor (D/A) architecture enables the electron transfer from the donor moiety to the acceptor moiety, leading to a strongly enhanced polarization and complete charge‐separated state.^[^ ^10^ ^]^ The charge transfer is accompanied by a perpendicularly twisted conjugated system lowering its energy and thus, leading to a red‐shift in the emission spectrum.^[^ ^11^ ^]^ As the occurrence of the TICT state and its energy level strongly depends on the environmental conditions, these fluorophores hold significant promise as fluorescent probes in terms of chemical sensing and reporting on microenvironmental polarities and viscosities.^[^ ^12^ ^]^ However, TICT‐based fluorescent probes generally suffer from low quantum yields since the excited TICT state is susceptible to non‐radiative decay pathways. In the quest of circumventing this drawback, chemists rely on chromophores with D/A architectures embedded in large aromatic *π*‐electron systems, for instance, pyrene, as they may retain their high quantum yields.^[^ ^13^ ^]^ To date, these tailor‐made fluorophores are used for cellular imaging,^[^ ^14^ ^]^ yet are not concurrently applied to report the physicochemical changes of the environment. An additional challenge is of course that, next to engineering the spectral characteristics and physicochemical properties, such multimodal molecular probes need to be easy‐to‐conjugate to specifically stain a targeted region of interest.^[^ ^15^ ^]^ This is also of particular relevance for the understanding of ex‐vitro soft matter systems, where there is increasing evidence that effects such as molecular crowding play important roles, for example in enhancing catalysis in protocellular environments.^[^ ^16^ ^]^ Those in turn are informative model systems for liquid/liquid phase segregated membraneless organelles that organize catalytic processes in living systems.^[^ ^17^ ^]^

**Figure 1:**
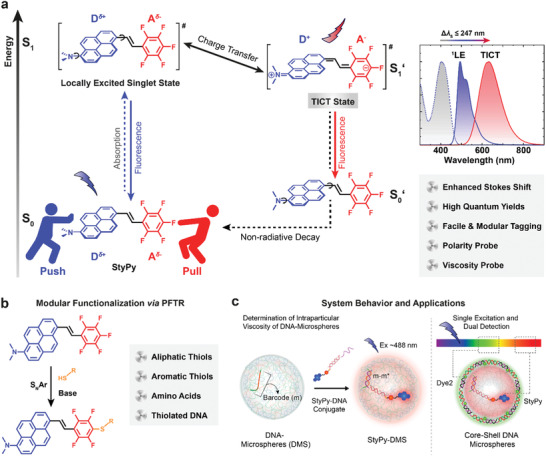
Rational design of a clickable and modular molecular sensor based on TICT. a) Scheme of the excited states of StyPy. The D/A architecture of StyPy enables the formation of a TICT state with a Stokes shift of Δ*λ*_S_ ≤ 247 nm and extraordinarily high quantum yields. b) Facile and modular functionalization of StyPy via S_N_Ar (*para*‐fluoro‐thiol reaction, PFTR). In the presence of a base, the PTFR proceeds orthogonal to other nucleophiles with aliphatic and aromatic thiols, with thiol‐containing amino acids, and with thiolated DNA. c) System behavior and applications. With the aid of the PFTR, the StyPy‐DNA conjugate (StyPy‐m*) is prepared that allows to selectively label DMS and measure viscosity and polarity. The enormous Stokes shift of StyPy enables us to detect two fluorophores, while using a single laser line as the excitation wavelength.

Inspired by the strategies mentioned above, herein, we introduce a multimodal molecular sensor based on a synthetically simple pentafluorostyryl‐aminopyrene (StyPy) push–pull structure which i) features high quantum yields of fluorescence, ii) undergoes TICT in the excited state showing an enormous Stokes shift (**Figure** **1**a), iii) enables to report local environmental constraints (polarity and viscosity), and iv) holds the modular applicability by facile conjugation via the metal‐free *para*‐fluoro‐thiol reaction (PFTR; Figure 1b)—a self‐reporting nucleophilic aromatic substitution (S_N_Ar).^[^ ^18^ ^]^ We describe details on the solvatochromic behavior in various solvents correlating the Stokes shifts to the environmental polarity, and further calibrate its TICT characteristics to the viscosity of the medium. Armed with molecular‐level understanding, we attach a single‐stranded DNA (ssDNA) via PFTR and incorporate it into different DNA hybrid mesostructures (Figure 1c). We measure the intraparticular viscosity in all‐DNA microspheres (DMS) as a function of the salt concentration, which regulates the crosslink exchange dynamics. Moreover, we image hydrophobic and hydrophilic compartments in DNA hybrid architectures, consisting of hydrophobic latex beads embedded in DMS, by confocal laser scanning microscopy (CLSM) using StyPy as a single dye in different environments via two separate detection windows while exciting with a single laser line. Finally, we take advantage of the enormous Stokes shift of StyPy to visualize two different parts of the DNA‐based morphologies using the StyPy conjugate and a classical fluorophore while employing a dual detection with a single laser excitation (Figure 1c).

## 2 Results and Discussion

### 2.1 Design Principles of StyPy

We focus on the rational design of a synthetically simple and modularly applicable molecular sensor, which can be readily incorporated into soft matter and biological systems as well as emits light with a substantial Stokes shift in response to its direct surroundings (Figure 1a). Thus, we designed a push–pull system, termed StyPy (6‐pentafluorostyryl‐1‐dimethylamino‐pyrene), that undergoes fast TICT upon excitation. The electron‐rich dimethylaminopyrenyl ring acts as an EDG, whereas the strongly electron‐deficient pentafluorophenyl ring acts as an EWG. In contrast to other push–pull TICT chromophores, we specifically selected pentafluorophenyl for its propensity to undergo efficient chemo‐selective S_N_Ar reactions in *para*‐position (PFTR; Figure 1b). Together both parts form a D/A fluorophore, which experiences a fast, intramolecular charge transfer from the donor part (highlighted in blue; Figure 1a) to the acceptor part (red) of the molecule in its excited state. This charge transfer induces an intramolecular twisting around two single bonds (highlighted by small arrows, top left).^[^ ^11a^ ^]^ Consequently, the fluorescence of the TICT state (S_1_′) is red‐shifted compared to the fluorescence of the locally excited singlet state (S_1_ or ^1^LE). At the same time, the highest occupied molecular orbital (HOMO) level of the TICT state (S_0_′) is elevated compared to the ground state (S_0_) of StyPy, which lowers the energy gap between S_1_′ and S_0_′, resulting in a further red‐shift of the fluorescence (enhanced Stokes shift). The equilibrium between these two excited states (S_1_ and S_1_′) often prompts in a dual fluorescence, which is a function of the local microenvironment of StyPy in terms of its polarity (i.e., the solvent) and the steric constraints of the molecule (i.e., the viscosity), rendering StyPy promising as a multiplexing molecular sensor.

**Figure 2:**
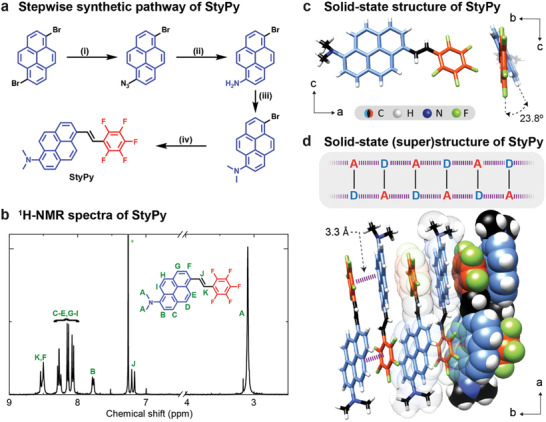
Synthesis and characterization of StyPy. a) Reaction conditions: i) NaN_3_, CuI, *N*,*N*′‐dimethyl‐ethylenediamine, sodium ascorbate, N_2_; ii) NaSH · *x*H_2_O, 38%; iii) MeI, K_2_CO_3_, N_2_, 100%; iv) pentafluorostyrene, Pd(OAc)_2_, NEt_3_, K_2_CO_3_, N_2_, 62%. b) ^1^H‐NMR spectrum of StyPy. The signals are assigned to the respective protons. The solvent residual signal is highlighted by a small star. c) Single crystal structure and d) packing of StyPy determined by X‐ray crystallography. The solid‐state structure shows that dimethylamino‐pyrenyl ring is twisted with a dihedral angle of 23.8° in comparison to the pentafluorostyryl ring. The packing of StyPy indicates the strong aromatic D/A interactions between both aromatic rings with a distance of 3.3 Å.

We synthesized StyPy following the illustrated scheme in four steps (**Figure** **2**a, conditions in the caption, details in Supporting Information). Starting from 1,6‐dibromo‐pyrene, the product is obtained with an overall yield of 23% and in good purity (>97%) according to ^1^H‐NMR spectroscopy and liquid chromatography‐mass spectrometry (Figure 2b). In order to gain more insight into the structural details of the molecular sensor, we performed single‐crystal XRD analysis (Figure 2c,d; see Table S1, Supporting Information, for crystallographic data). The solid‐state structure of StyPy shows that the pyrenyl ring is twisted in comparison to the pentafluorostyryl ring with a dihedral angle of 23.8° (Figure 2d). The two fluorine atoms in ortho‐position to the central double‐bond in conjunction with the sterically demanding pyrenyl ring force the molecule into a “pretwisted” conformation in solid‐state. Moreover, the dimethylamino group is twisted with respect to the pyrenyl ring (26.3°) as well. According to previous TICT studies, a pretwisted conformation in the ground state stabilizes the TICT state upon excitation and thus favors it, which generally leads to a larger red‐shift in fluorescence.^[^ ^11^, ^12^, ^19^ ^]^ The solid‐state (super)structure of StyPy exhibits aromatic D/A interactions between the dimethylaminopyrenyl and the pentafluorophenyl ring with an alternating binary arrangement in a head‐to‐tail fashion (Figure 2d). The distance between the two *π*‐surfaces is approximately 3.3 Å, which coincides with conventional aromatic D/A interactions.^[^ ^20^ ^]^ These preliminary characterization methods already illustrate a pretwisted conformation of StyPy in the solid‐state as well as the strong aromatic D/A interactions in StyPy, which further motivates the analysis of its properties as TICT‐based fluorescent probe.

### 2.2 Functionalization of StyPy via PFTR

**Figure 3:**
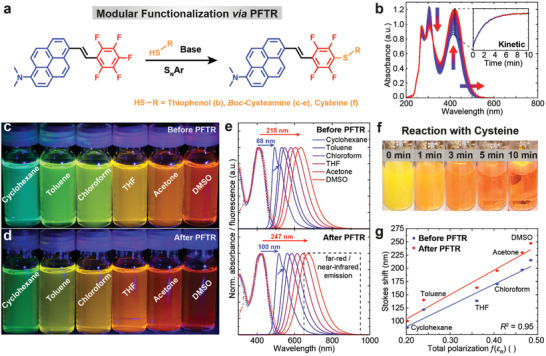
Spectroscopic characteristics of StyPy before and after the *para*‐fluoro‐thiol reaction (PFTR). a) Reaction scheme of the PFTR (S_N_Ar). b) Time‐dependent UV/Vis spectra (*n* = 121). StyPy (50 µm) and thiophenol react in DMSO and DBU at 37 °C over the course of 10 min. The inset shows the absorbance at 425 nm over time, indicating the kinetic profile of the PFTR. Photographs of c) pristine StyPy (50 µm) and d) *post*‐functionalized StyPy (50 µm) in different solvents irradiated with a blue LED (431 ± 7 nm). e) Normalized UV/Vis (dashed lines, 50 µm) and fluorescence (solid lines, 50 µm) spectra before (top) and after functionalization (bottom) with respect to the solvent. Before the functionalization, the Stokes shift Δ*λ*_S_ varies from 88 nm (cyclohexane) to 215 nm (DMSO). After functionalization, it differs from 100 nm (cyclohexane) to 247 nm (DMSO). f) Reaction of StyPy (5 mg·mL^−1^) with cysteine at 37 °C in the presence of DBU. The reaction proceeds from a turbid yellow dispersion (left) to a clear orange solution (right) within 10 min in DMF/water. g) Correlation between the polarity and the Stokes shift. The Stokes shift approximately depends on the solvent polarity, indicated by the total polarization *f*(*ε*_R_), in a linear manner.

Before we analyze the characteristics of StyPy as a molecular sensor, we demonstrate the versatility of the PFTR on the small molecule level and how easily the conjugation to thiol‐bearing molecules can be monitored (**Figure** **3**a). In the presence of a base, the PFTR occurs with nearly quantitative yields using simple aliphatic and aromatic thiols resulting in a thioether of StyPy. While the nucleophilic attack of thiols occurs readily at RT, primary amines and hydroxy groups need high temperatures (>70 °C) as well as strong bases (e.g., KOH).^[^ ^21^ ^]^ Hence, the PFTR proceeds under comparably mild conditions and orthogonal to other nucleophiles, specifically targeting accessible thiols.^[^ ^22^ ^]^ The progress of the reaction is macroscopically visible by a color change of the reaction mixture as seen for instance for the reaction of StyPy (1.0 equiv., 5 mg·mL^−1^) with the thiol‐containing amino acid cysteine (1.0 equiv.) in the presence of the base DBU (1,8‐diazabicyclo(5.4.0)undec‐7‐ene, 2.0 equiv.). The images in Figure 3f show that the reaction proceeds from a turbid yellow dispersion (left) to a clear orange solution (right) within 10 min at 37 °C. The red‐shift, which occurs during the reaction, stems from the fact that the thioether acts as an additional EDG forming a D/A/D *π*‐electron system that further lowers the energy gap between the HOMO and the lowest unoccupied molecular orbital (LUMO). This spectral change allows us to easily track the progress of the reaction by UV/Vis and fluorescence spectroscopy. While the red‐shift is already macroscopically visible using StyPy and cysteine, a more significant change occurs in the case of an aromatic thiol, such as thiophenol, which further extends the core conjugation. Figure 3b displays time‐dependent UV/Vis spectra of the reaction of StyPy (1.0 equiv., 50 µm) with thiophenol (1.0 equiv.) in presence of DBU (2.0 equiv.) at 37 °C. The spectra evolve from the spectrum of the pristine StyPy (blue) to the StyPy‐thiophenol‐adduct (red) with a discernible isosbestic point at 322 nm. The isosbestic point indicates the clean transformation from StyPy to the *para*‐substituted StyPy thioether, which further confirms the promising characteristic of the PFTR to specifically target accessible thiols in a selective fashion. The inset shows the absorbance at 425 nm plotted against the reaction time (Figure 3b). Assuming pseudo‐first‐order kinetics, the kinetic analysis yields a half‐life of 1.40 ± 0.01 min at 37 °C in DMSO and is practically complete within 8–10 min. Moreover, the pentafluorophenyl ring allows to quantitatively track the PFTR via ^19^F‐NMR spectroscopy. The three signals of the mono‐substituted perfluorinated phenyl ring disappear over time, and two signals appear, indicating the *para*‐substitution pattern of the StyPy thioether (Figure S1, Supporting Information). The fast reaction of StyPy, while explicitly targeting accessible thiols, paves the way towards labeling (bio)macromolecules of interest, such as thiolated ssDNA.

### 2.3 StyPy as a Polarity Probe

UV/Vis and fluorescence measurements in correlation with the solvent polarity provide a first simple indication whether StyPy undergoes TICT in its excited state. Thus, we analyzed the absorption and emission characteristics of StyPy in six different solvents, ranging from apolar cyclohexane to polar DMSO, and compared it to the functionalized StyPy after coupling StyPy to Boc‐protected cysteamine via PFTR (Figure 3a). The UV/Vis spectra (dashed lines, Figure 3e) hardly change as a function of the solvent polarity for both compounds. Consequently, the ground states of both dyes are nearly unaffected by the solvent polarity and exhibit no solvatochromism in terms of their absorptivity. In contrast, the emission spectra of both fluorophores strongly shift with respect to the solvent polarity (solid lines; Figure 3e). In DMSO, both the pristine and the *post*‐functionalized StyPy exhibit a strongly red‐shifted emission with a maximum of *λ*_em_ = 633 nm (top) and *λ*_em_ = 672 nm (bottom), which corresponds to a Stokes shift of Δ*λ*_S_ = 215 nm and Δ*λ*_S_ = 247 nm, respectively. The high polarizability of DMSO leads to a stabilization of the TICT state, which lowers its energy tremendously. Therefore, the resulting fluorescence red‐shifts with a broad emission into the far‐red/NIR region (highlighted by a dashed rectangle). In cyclohexane, StyPy and the StyPy thioether exhibit the least red‐shifted fluorescence with emission maxima of *λ*_em_ = 494 nm (top) and *λ*_em_ = 516 nm (bottom), corresponding to a Stokes shift of Δ*λ*_S_ = 88 nm and Δ*λ*_S_ = 100 nm, respectively. In the least polar solvent cyclohexane, fluorescence from the locally excited singlet state (^1^LE) is favored over the TICT state (shoulders highlighted by small arrows; Figure 3e). The occurrence of a dual fluorescence in cyclohexane underscores that StyPy undergoes TICT upon excitation in addition to the yet dominant singlet state emission. The singlet state emission however disappears for the more polar solvents due to better stabilization of the TICT state in those. To vividly illustrate the influence of the solvent polarity on the emission, we captured photographs of the pristine and the functionalized StyPy, dissolved in different solvents, while irradiating them with a blue LED (Figure 3c,d). Before the PFTR, the fluorescence gradually changes from turquoise in cyclohexane to dark orange in DMSO. After the PFTR, the overall emission is further red‐shifted because of the D/A/D architecture, ranging from green in cyclohexane to dark red in DMSO. To analyze the dependency of the Stokes shift on the solvent polarity, we calculated the total polarization *f*(*ε*_R_) according to the Kirkwood Equation (1) using the relative permittivity *ε*_R_ of the solvents.^[^ ^23^ ^]^

(1) $$f\left(\varepsilon_{R}\right)\;=\;\frac{\varepsilon_{R\;}-\;1}{2\;\times \;\varepsilon_{R\;}+\;1}$$

The plot of Δ*λ*_S_ as a function of *f*(*ε*_R_) shows a linear correlation between the Stokes shift and the total polarization for both the pristine StyPy (blue; Figure 3g) and the functionalized StyPy (red) with a correlation coefficient of *R* ^2^ = 0.95 in both cases. This strong correlation unambiguously demonstrates StyPy to be a promising fluorescent probe that reports on the local polarity via the emission maximum *λ*_em_.

### 2.4 Fluorescence Quantum Yields *Φ*_Fl_

Fluorophores undergoing TICT upon excitation along with large Stokes shifts feature the critical advantage that the absorbance and fluorescence barely overlap, which drastically minimizes self‐absorption and therefore autofluorescence. However, these fluorophores generally suffer from low quantum yields of fluorescence *Φ*_Fl_,^[^ ^11b^ ^]^ since the TICT states are prone to non‐radiative decay pathways (Figure 1a). Konishi and coworkers reported pyrene analogues of Prodan (2‐propionyl‐6‐dimethylaminonaphthalene), which undergo TICT upon excitation while retaining the generally high quantum yields of pyrene derivatives.^[^ ^13b^ ^]^ This escape from the classical dilemma motivated us to analyze the fluorescence quantum yields of StyPy in different solvents (for experimental details see Figures S2 and S3, Supporting Information). Indeed, the unfunctionalized StyPy features extraordinarily high quantum yields of fluorescence with *Φ*_Fl_ > 0.92 in all six organic solvents (**Table** **1**). The comparison of the quantum yields of StyPy before and after the PFTR reveals that the quantum yields decrease slightly in acetone (*Φ*_Fl_ = 0.80) and DMSO (*Φ*_Fl_ = 0.76), but otherwise remain high (*Φ*_Fl_ > 0.94). Moreover, the sensitivity of a fluorescent probe is described by its brightness, which is defined as the product of the extinction coefficient and the quantum yield (*ε* · *Φ*_Fl_).^[^ ^4^ ^]^ Table 1 shows that the brightness of StyPy before and after the PFTR is above 2.0 × 10^4^ m·cm^−1^ in all organic solvents with maximum brightness in chloroform close to 3.0 × 10^4^ m cm^−1^ after conjugation. This is an improvement of up to 100% compared to the widely applied solvatochromic dye Prodan (approx. 1.5 × 10^4^ m cm^−1^ in ethanol).^[^ ^13b^ ^]^ Furthermore, we determined the photostability of the functionalized StyPy in DMSO. To this end, we continuously irradiated a solution (50 µm) of it with a blue high‐intensity LED (431 ± 7 nm, 10 mW cm^−2^) and recorded fluorescence spectra in intervals of 10 min (Figure S4, Supporting Information). Based on the integration of the fluorescence intensity, the functionalized StyPy shows a photoinduced fatigue with a half‐life of approximately 18 h, assuming first‐order kinetics of photobleaching. The strong brightness in conjunction with the highly tunable Stokes shift (ΔΔ*λ*_S_ ≤ 147 nm), the robust photostability and the facile conjugation renders StyPy particularly promising as a targeted polarity probe.

**Table 1:** Spectroscopic characteristics of StyPy before and after PFTR

	Before functionalization					After functionalization				
Solvent	*ε*_425 nm_ [m cm^−1^]	*λ*_em_ [nm]	Δ*λ*_S_ [nm]	*Φ*_Fl_ ()	*ε* × *Φ*_Fl_ [m cm^−1^]	*ε*_425 nm_ [m cm^−1^]	*λ*_em_ [nm]	Δ*λ*_S_ [nm]	*Φ*_Fl_ ()	*ε* × *Φ*_Fl_ [m cm^−1^]
Cyclohexane	2.52 × 10^4^	494	88	0.92	2.3 × 10^4^	2.85 × 10^4^	516	100	0.96	2.7 × 10^4^
Toluene	2.61 × 10^4^	532	122	≈1.00	2.6 × 10^4^	2.82 × 10^4^	562	140	≈1.00	2.8 × 10^4^
Chloroform	2.68 × 10^4^	549	138	≈1.00	2.7 × 10^4^	2.89 × 10^4^	585	163	≈1.00	2.9 × 10^4^
THF	2.29 × 10^4^	579	170	0.98	2.2 × 10^4^	2.63 × 10^4^	614	196	0.94	2.5 × 10^4^
Acetone	2.53 × 10^4^	604	197	0.92	2.3 × 10^4^	2.97 × 10^4^	645	230	0.80	2.4 × 10^4^
DMSO	2.45 × 10^4^	633	215	0.92	2.3 × 10^4^	2.66 × 10^4^	672	247	0.76	2.0 × 10^4^
Water^a)^	‐	‐	‐	‐	‐	1.83 × 10^4^	649	241	0.28	5.1 × 10^3^

*ε*_425 nm_, molar extinction coefficient; *λ*_em_, emission maximum; Δ*λ*_S_, Stokes shift; *Φ*_Fl_, quantum yield of fluorescence; *ε × Φ*_Fl_, brightness

**Figure 4:**
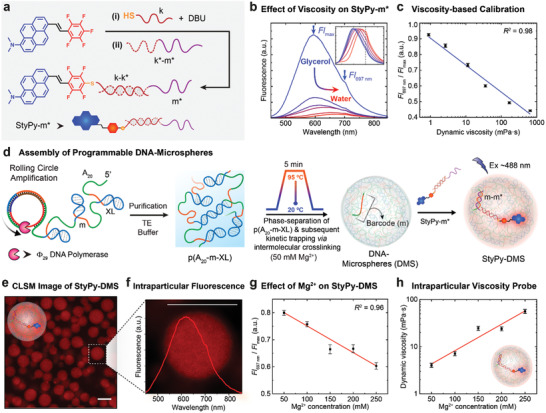
StyPy as a viscosity probe in all‐DNA microspheres. a) Scheme to prepare StyPy‐m*. b) Fluorescence spectra of StyPy‐m* (2 µm) as a function of the glycerol/water ratio. The fluorescence intensity decreases as well as the emission red‐shifts with a higher fraction of water (curved arrow). The inset shows the corresponding normalized emission. c) Viscosity‐based calibration. The ratio (*Fl*_697 nm_/*Fl*_max_) depends linearly on the logarithmic dynamic viscosity (lg *η*). d) Preparation of all‐DNA microspheres. The polymer p(A_20_‐m‐XL) is prepared via rolling circle amplification, which phase‐separates upon heating in the presence of Mg^2+^ (50 mm). The binodal phase separation is kinetically trapped by intermolecular crosslinks. e) CLSM image of StyPy‐labeled DMS. The incorporation of StyPy into the DNA matrix enables a facile imaging without the need for a strong laser intensity and/or a high gain (scale bar = 3.0 µm). f) Intraparticular fluorescence of StyPy‐m*. The fluorescence spectrum shows a broad emission in the far‐red/near‐infrared regime with a shoulder at 697 nm. g) Effect of Mg^2+^ on the emission of StyPy immobilized in DMS. The dimensionless parameter (*Fl*_697 nm_/*Fl*_max_) decreases linearly with an increasing Mg^2+^ concentration, thus indicating greater crosslinking density. h) StyPy as an intraparticular viscosity probe. The calibration in (c) is used to calculate the dynamic viscosity from the ratio of *Fl*_697 nm_ and *Fl*_max_. Hence, the viscosity increases with an increasing Mg^2+^ concentration.

^a)^ Measured with StyPy‐m* (see Figure 4a).

### 2.5 StyPy as a Viscosity Probe

To move towards applications in DNA nanoscience, we focused on the second important characteristic, which is the performance as a spectral viscosity probe. With the aid of the PFTR, we functionalized thiolated ssDNA with StyPy. This DNA‐StyPy conjugate was subsequently hybridized with a complementary ssDNA containing a specific DNA barcode sequence (m*) to form StyPy‐m* (**Figure** **4**a; Figure S5, Supporting Information). StyPy‐m* allows for later hybridization with the barcode m to selectively label DMS. We analyzed the emission characteristics of StyPy‐m* in glycerol/water mixtures that have tunable dynamic viscosity *η* from pure water (*η* = 0.89 mPa s) to 98 vol% glycerol (*η* = 653 mPa s; Figure 4b).^[^ ^24^ ^]^ The fluorescence spectra show a stronger blue‐shift as well as a higher intensity with an increasing ratio of glycerol (highlighted by a curved arrow). Since glycerol has a similar polarity as DMSO as indicated by the dielectric constants (*ε*_R_ = 47.0 vs *ε*_R_ = 46.7) the large blue‐shift of 80 nm (from 672 to 592 nm) does not majorly stem from the small polarity mismatch between water and glycerol, but from the increased viscosity. To eliminate effects of spectral shifts and lower quantum yields in water (Table 1) due to aggregation‐induced quenching^[^ ^25^ ^]^ and opening of non‐radiative decay pathways due to hydrogen binding with water,^[^ ^4^ ^]^ we determined a dimensionless parameter. This parameter is based on the shoulder, which appears at a wavelength of 697 nm in the fluorescence spectra of StyPy‐m*, and which is distinctly more pronounced for water than for glycerol (inset; Figure 4b). Plotting the fluorescence intensity at 697 nm (*Fl*_697 nm_) divided by the maximum intensity *Fl*_max_ (highlighted by small arrows) against the logarithmic dynamic viscosity (lg *η*) yields a distinct linear correlation (Figure 4c). This linear correlation unambiguously proves that StyPy reports on the dynamics of its microenvironment. Hence, StyPy unites both the characteristics of a polarity probe as well as of a viscosity probe.

### 2.6 StyPy as a Viscosity Probe in Colloidal DNA Mesostructures

Having established the viscosity‐based calibration, we applied these probes to understand DMS formed by temperature‐promoted phase segregation in terms of their interior viscosity. Such an understanding is for instance of high importance for the field of coacervate droplets, molecular crowding, and catalysis in coacervates, where often only small amounts are available in the colloidal state that cannot be studied using classical rheology. For instance, we recently showed that the catalytic efficiency of an encapsulated artificial metalloenzyme for a ring‐closing metathesis reaction could be enhanced due to the biomacromolecular crowding at the core of a protocell.^[^ ^17^ ^]^ Thus, we first prepared a ssDNA multiblock polymer, namely p(A_20_‐m‐XL), via rolling circle amplification (Figure 4d). p(A_20_‐m‐XL) is equipped with three repeating blocks: an adenine_20_ unit (A_20_), the barcode (m) sequence (which is complementary to m* of StyPy‐m*), and a palindromic sequence (XL). The DNA‐microspheres form when a mixture of p(A_20_‐m‐XL) is subjected to a rapid heating ramp to 95 °C in presence of 50 mm Mg^2+^.^[^ ^26^ ^]^ Critically, at least ≈50 mm Mg^2+^ has to be present to allow for the particle formation. During the heating ramp, the p(A_20_‐m‐XL) phase‐separates into coacervates in the presence of Mg^2+^ above its cloud point temperature (*T*_cp_ = 60 °C). During cooling, these droplets are kinetically trapped via intraparticular duplex‐type crosslinking of the self‐complimentary XL domains giving rise to perfectly spherical DMS. Subsequently, the DMS were labeled with StyPy‐m* by selectively targeting the complementary barcodes m. Hence, the particles can be visualized using the imaging capabilities of the probes via CLSM (Figure 4e). Figure 4f displays the corresponding fluorescence spectrum of StyPy‐m* immobilized inside the DNA matrix with a broad emission in the far‐red/NIR regime and the shoulder at 697 nm, which allows to determine the interior viscosity.

Since Mg^2+^ is in fact the key ingredient to ensure that the p(A_20_‐m‐XL) undergoes temperature‐induced phase segregation and particle formation, and since it is known that it effectively strengthens DNA binding and interactions (Mg^2+^ increases DNA melting), we probed the viscosity of the StyPy‐DMS by fluorescence spectroscopy as a function of the Mg^2+^ concentration (50–250 mm; Figure S6, Supporting Information). The plot of the ratio of *Fl*_697 nm_ and *Fl*_max_ against the Mg^2+^ concentration indicates a linear relationship (Figure 4g). Consequently, a higher amount of the Mg^2+^ increases the crosslinking density and lowers the crosslink exchange dynamics inside the DMS, which consequently increases the dynamic viscosity. With the aid of the previous viscosity‐based calibration (Figure 4c), the ratio (*Fl*_697 nm_/*Fl*_max_) can be converted into the dynamic viscosity, which subsequently illustrates the linear dependence of the dynamic viscosity on the Mg^2+^ concentration (Figure 4h)—a relevant parameter in terms of molecular crowding and dynamics, which would be hard to access using other means. In order to verify that this effect is indeed induced by the viscosity, and not by Mg^2+^ itself, we performed control experiments for StyPy‐m* in solution as a function of the Mg^2+^ concentration (50–250 mm; Figure S7, Supporting Information). Since there is only a negligible change, the large changes in the DMS are indeed related to the local viscosity.

### 2.7 StyPy as a Probe for Compartmentalization

**Figure 5:**
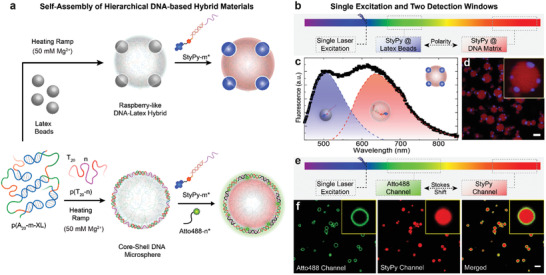
StyPy as a multimodal probe to simultaneously sense polarity or compartments using single laser excitation. a) Scheme to prepare two different DNA microspheres: i) Raspberry‐like DNA hybrids (top) and ii) core‐shell DNA microspheres (bottom). b) Scheme of the single excitation of StyPy located at different compartments. c) Fluorescence spectrum of StyPy‐m* (2 µm) embedded in raspberry‐like DNA hybrids. The spectrum shows a superposition of the spectrum of StyPy assembled at the latex beads (blue) and of StyPy inside the DNA matrix (red). d) CLSM image of raspberry‐like DNA hybrids. The non‐fluorescent latex beads are brighter than the DNA matrix since the brightness of StyPy is increased in the hydrophobic environment due to the suppression of solvent‐mediated relaxation (scale bar = 3.0 µm). e) Scheme of single laser excitation. Two dyes are excited with one laser line while their emission is detected in different regimes of the spectrum. f) CLSM images of core‐shell DNA protocells. The core‐shell architecture allows to selectively stain the core (StyPy) and the shell (Atto488) due to the barcodes m* and n*, respectively. The large Stokes shift of StyPy enables to detect both dyes in two different channels (scale bar = 3.0 µm).

Furthermore, we used StyPy as a probe to report on hydrophobic and hydrophilic compartments. In this context, we prepared positively charged, non‐fluorescent latex beads (*r*_TEM_ = 102 ± 4 nm; Figure S8, Supporting Information) made of TFEMA (2,2,2‐trifluoroethyl methacrylate) and mixed them with the ssDNA polymer p(A_20_‐m‐XL). Upon applying a heating ramp to the dispersion in presence of Mg^2+^, raspberry‐like hybrid microspheres are formed, which were subsequently labeled with StyPy‐m* (top; **Figure** **5**a). The corresponding emission spectrum clearly shows a superposition of two emission spectra—one of StyPy immobilized inside the DNA matrix (highlighted in red, *λ*_em_ = 635 nm; Figure 5c) and one of StyPy assembled at the interface between the latex beads and the DNA matrix (highlighted in blue, *λ*_em_ = 504 nm). This confirms that StyPy is able to selectively probe different environments inside the DNA raspberry‐like hybrids. Note, that the emission maximum of 504 nm, corresponding to the PTFEMA part, is further blue‐shifted than the emission of StyPy dissolved in the least polar solvent cyclohexane (*λ*_em_ = 516 nm, *ε*_R, cyclohexane_ = 2.0; Table 1). This phenomenon is a consequence of the characteristics of StyPy to probe both the polarity (*ε*_R, PTFE_ = 2.1) and the viscosity of the solid fluorinated latex beads. To spatially resolve the superposition of the emission spectra of StyPy assembled at the interface and of StyPy immobilized inside the DNA matrix, we acquired CLSM images (Figure 5d). We set two different detection windows (495–530 nm and 675–740 nm; Figure 5b) to selectively image StyPy in the different environments of the raspberry‐like hybrids. The corresponding CLSM image vividly illustrates how StyPy—as a single fluorophore—reports on different environments and differentiates between hydrophobic and hydrophilic compartments. In contrast, when we used the exact same raspberry‐like hybrid particles, but stained them with the hydrophilic Atto647‐m* dye, the latex beads remain non‐fluorescent (Figure S9, Supporting Information). Hence, StyPy extends beyond conventional dyes to report on environmental constraints due to lack of adsorption on the beads.

### 2.8 Single Excitation and Two Detection Windows in Core‐Shell DNA Protocells

Finally, we exploited the large Stokes shift of StyPy to demonstrate advances in multi‐fluorophore imaging by imaging a classical dye (Atto488) and our StyPy with a single laser line (488 nm), while detecting the subsequent emission at two different spectral windows (Figure 5e). To this end, we prepared core‐shell DNA protocells made of p(A_20_‐m‐XL) and p(T_20_‐n) (thymine_20_), whereas the latter does not undergo phase separation upon heating and forms a shell around the p(A_20_‐m‐XL) core during the cooling step via A_20_‐T_20_ hybridization (bottom; Figure 5a).^[^ ^26^ ^]^ Subsequently, the cores and the shells were selectively stained using the complementary barcodes m (core, by StyPy‐m*) and n (shell, by Atto488‐n*). Upon exciting these core‐shell DNA protocells with a single 488 nm laser line, clearly spectrally separated emission in the green and in the red/far‐red regime can be detected. This is particularly interesting for extremely fast, time resolved CLSM imaging, because a sequential scanning is obsolete in this case. The corresponding CLSM images show that the shell (green channel; Figure 5f) and the core (red channel) are clearly distinguishable from each other in the merged image.

## 3 Conclusion

We introduced the molecularly engineered fluorescent probe StyPy which is modularly and readily processable via the PFTR and combines the properties of a polarity as well as a viscosity probe. To exploit these features on the material scale, we prepared the DNA‐conjugate StyPy‐m* that enables to selectively label raspberry‐like hybrid DNA particles consisting of non‐fluorescent latex beads embedded in DMS. Here, StyPy reports on the different hydrophobic and hydrophilic compartments based on the superposition of two emission spectra. Considering the amphiphilic environments of cell membranes, this feature is particularly interesting, because it may give direct insights into changes in terms of their polarity and/or viscosity of various compartments as a function of external or internal stimuli. Moreover, we investigated the emission of StyPy‐m* in solution with respect to the viscosity and found a linear correlation between the logarithmic dynamic viscosity (lg *η*) and the dimensionless parameter (*Fl*_697 nm_/*Fl*_max_). With the aid of this calibration, we probed the intraparticular viscosity of DMS and how it changes with respect to the Mg^2+^ concentration. As processes, such as metabolism, signaling and transport are mediated by the intracellular viscosity, StyPy may indicate slight changes here, while featuring a broad emission in the far‐red/NIR region. Finally, StyPy possesses a D/A architecture with an extended *π*‐electron system that results in extraordinary quantum yields of fluorescence. The push–pull system in conjunction with its pretwisted conformation leads to an enormous (Δ*λ*_S_ ≤ 247 nm) and highly tunable (ΔΔ*λ*_S_ ≤ 159 nm) Stokes shift with nearly no overlap between absorbance and emission. On the material level, the enormous Stokes shift enables to detect multiple dyes that are excited with a single laser line. This is particularly interesting for extremely fast, time resolved CLSM imaging, because a sequential scanning is obsolete in this case.

Importantly, we believe that designing such molecular sensors will broaden the horizon to closely quantify local environmental constraints in artificial or biological complex architectures with their tunable fluorescence. Moreover, these fundamental studies of StyPy conjugates from the molecular to material level are of great importance in the field of biomedical molecular imaging and diagnostics because our approach allows facile and modular labeling of (bio)macromolecules of interest (e.g., DNA and amino acids), sensing of local microenvironmental changes (e.g., polarity and viscosity), and the inhibition of autofluorescence based on self‐excitation (enhanced Stokes shift with no overlap).

## 4 Crystallographic Data

CCDC 1986004 contains the supplementary crystallographic data for this paper. These data can be obtained free of charge from The Cambridge Crystallographic Data Centre via www.ccdc.cam.ac.uk/structures.

## Conflict of Interest

The authors declare no conflict of interest.

## Supporting information

Supporting Information
