# Supplementary material for: A Modular Fluorescent Probe for Viscosity and Polarity Sensing in DNA Hybrid Mesostructures
Source: Adv Sci (Weinh). 2020 Dec 23;8(5):2003740. doi: 10.1002/advs.202003740 (PMC7927630; doi:10.1002/advs.202003740)
Supplement: Supplementary file 1 — Supporting Information [file ADVS-8-2003740-s001.pdf]

© 2020 Wiley-VCH GmbH

ADVANCED SCIENCE
Open Access

# Supporting Information

for *Adv. Sci.,* DOI: 10.1002/advs.202003740

# A Modular Fluorescent Probe for Viscosity and Polarity Sensing in DNA Hybrid Mesostructures

*Simon Ludwanowski, Avik Samanta, Sebastian Loescher, Christopher Barner-Kowollik, and Andreas Walther**

# Supporting Information

# A Modular Fluorescent Probe for Viscosity and Polarity Sensing in DNA Hybrid Mesostructures

Simon Ludwanowski,[1-3] Avik Samanta,[1-3] Sebastian Loescher,[1-3] Christopher Barner-Kowollik,[5,6] Andreas Walther[1-4*]

---

[1]Institute for Macromolecular Chemistry, University of Freiburg, Stefan-Meier-Straße 31, 79104 Freiburg, Germany.

[2]Freiburg Materials Research Center (FMF), University of Freiburg, Stefan-Meier-Straße 21, 79104 Freiburg, Germany.

[3]Freiburg Center for Interactive Materials and Bioinspired Technologies (FIT), University of Freiburg, Georges-Köhler-Allee 105, 79110 Freiburg, Germany.

[4]Cluster of Excellence livMatS @ FIT – Freiburg Center for Interactive Materials and Bioinspired Technologies, University of Freiburg, Georges-Köhler-Allee 105, D-79110 Freiburg, Germany

[5]School of Chemistry, Physics and Mechanical Engineering, Queensland University of Technology (QUT), 2 George Street, Brisbane, 4000 QLD, Australia.

[6]Macromolecular Architectures, Institute for Technical Chemistry and Polymer Chemistry, Karlsruhe Institute of Technology (KIT), Engesserstr. 18, 76128 Karlsruhe, Germany

*Correspondence to: andreas.walther@makro.uni-freiburg.de

## Materials

All chemicals were bought and used without further purification: 1,6-Dibromopyrene (Synthonix, 98.0 %), copper iodide (Merck, for synthesis), sodium ascorbate (Sigma Aldrich, >99,0 %), *N*,*N'*-dimethyl-ethylenediamine (DMEDA, Alfa Aesar, 95.0 %), sodium azide (Abcr, >99.5 %), sodium hydrosulfide hydrate (Sigma Aldrich), potassium carbonate (Abcr, 99.0 %), iodomethane (Abcr, 99.5 %), palladium(II) acetate (Sigma Aldrich, 97.0 %), triphenylphosphine (Roth, >99.5 %), pentafluorostyrene (Abcr, 98 %), 1,8-diazabicyclo(5.4.0)undec-7-ene (DBU, TCI, >98.0 %), tert-Butyl N-(2-mercaptoethyl)carbamate (*Boc*-protected cysteamine, Sigma Aldrich, 97 %), *L*-cysteine (Merck, >99.0 %), thiophenol (Alfa Aesar, >99.0 %), tris(2-carboxyethyl)phosphine hydrochloride (TCEP · HCl, Abcr, 99.0 %), 2,2,2-trifluoroethyl methacrylate (TFEMA, Abcr, 99 %), divinylbenzene (DVB, Sigma Aldrich, 80 %), *N*-isopropylacrylamide (NIPAM, Sigma Aldrich, 97 %), 2,2′-azobis(2-methylpropionamidine) dihydrochloride (V50, Abcr, 96 %).

All ssDNA oligomers were purchased from Integrated DNA Technologies (IDT) as listed below in Table S2. The enzymes $T_4$ ligase (low concentration 2 $U \cdot \mu L^{-1}$), Exonuclease I (40 $U \cdot \mu L^{-1}$), and Exonuclease III (200 $U \cdot \mu L^{-1}$) were purchased from Lucigen. Inorganic pyrophosphatase (2 $U \cdot \mu L^{-1}$) and $\Phi_{29}$ polymerase (10 $U \cdot \mu L^{-1}$) were purchased from New England Biolabs and Promega, respectively. Deoxynucleotide triphosphate (dATP, dTTP, dGTP and dCTP) 100-110 mM, 1 mL were purchased from Jena Bioscience.

## Instrumentation

### NMR spectroscopy

NMR measurements were conducted on 400 MHz (9.3 T) Bruker Ascent 400 spectrometer. Coupling constants $J$ and chemical shifts $\delta$ are displayed in Hz and in ppm, respectively. The signals of deuterated solvents were used as internal standards.

### Transmission electron microscopy (TEM)

TEM images were acquired using a FEI Talos 120C at 120 kV operating voltage.

### Liquid chromatography mass spectrometry (LC-MS)

LC-MS measurements were performed on an UltiMate 3000 UHPLC System (Dionex, Sunnyvale, CA, USA) consisting of a pump (LPG 3400SZ), autosampler (WPS 3000TSL) and a temperature-controlled column compartment (TCC 3000). Separation was performed on a C18 HPLC column (Phenomenex Luna 5 µm, 100 Å, 250 × 2.0 mm) operating at 40 °C with water/acetonitrile as eluents. Spectra were recorded on an LTQ Orbitrap Elite mass spectrometer (Thermo Fisher Scientific, San Jose, CA, USA) equipped with a HESI II probe.

### UV/Vis and fluorescence spectroscopy

UV/Vis and fluorescence measurements were carried out on an QE Pro from Ocean Optics equipped with the light source DH-2000-BAL and the temperature-controlled cuvette holder qpod 2e™ from Quantum Northwest for fiber optic spectroscopy, which enables to successively record UV/Vis spectra in 180° and fluorescence spectra in 90° (Figure S2). Time-resolved measurements were acquired using self-written MatLab scripts.

### Plate reader

Fluorescence spectroscopic measurements with the DNA-microspheres were acquired using the Tecan Spark plate reader in top mode. The black 384 well plates from Costar Corning were used in which every well was filled with 30 µL of solution. The plate was kept at 25 °C during all the measurements.

### Tunable NKT Photonics Laser

Samples were irradiated with an EXR-15 supercontinuum white light laser from NKT Photonics. The monochromatic irradiation wavelength was generated using the SuperK EXTEND-UV filter box from NKT Photonics.

### LED set-up

Samples were irradiated with a fiber-coupled LED from Mightex (420 nm: 431 ± 7 nm, 310 mW).

### pH measurements

pH measurements were carried out on a 907 Titrando from Metrohm.

## Statistical analysis

Unless otherwise noted, all measurements were performed in triplicates ($n = 3$) to calculate the mean ± standard deviation. Linear regressions were calculated using OriginPro 2018G. Gaussian and mono-exponential fits were obtained by the Levenberg-Marquardt algorithm using the non-linear Origin Basic Functions (Gauss and ExpDec1) implemented in OriginPro 2018G. Fluorescence intensities were determined by the integration of the emission spectra using the tool curve_integ implemented in OriginPro 2018G. UV/Vis and fluorescence spectra were normalized to the absorption and the emission maximum, respectively.

## Synthesis

### Synthesis of 1-amino-6-bromopyrene

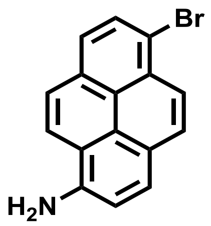


1,6-Dibromo-pyrene (1.00 g, 2.80 mmol, 1.00 eq.), CuI (56.1 mg, 0.295 mmol, 10.5 mol-%) and sodium ascorbate (33.4 mg, 0.169 mmol, 6.0 mol-%) were dispersed in THF (70 mL) and nitrogen-purged (15 min). *N*,*N*'-dimethyl-ethylenediamine (DMEDA, 38.5 mg, 0.437 mmol, 15.6 mol %) dissolved in THF (10 mL) was added to the reaction mixture. Subsequently, $NaN_3$ (195 mg, 3.00 mmol, 1.07 eq.) dissolved in water (7 mL) was added dropwise and the reaction mixture was refluxed for 15 hours. THF was removed *in vacuo* and the residue was washed twice with water (2 x 30 mL). The aqueous phase was removed *via* centrifugation and subsequently, by lyophilization yielding 928 mg raw product. The raw product was dissolved in THF (95 mL) and mixed with NaSH · $H_2O$ (2.97 g, 40.1 mmol, 13.8 eq.) dissolved in water (9.5 mL). The red solution was refluxed for 3 hours. Subsequently, it was cooled to RT and mixed with chloroform (40 mL) and water (40 mL) and the aqueous phase was extracted twice with chloroform (2 x 40 mL). The combined organic phases were dried *in vacuo* and the residue was purified by flash column chromatography ($SiO_2$) using pure DCM as the eluent. Yield: 312 mg (37.6 %). $^1$H-NMR ($CDCl_3$, 400 MHz, 25 °C): $\delta$ = 8.16 (dd, *J* = 8.7, 5.5 Hz, 2H), 8.02 (dd, *J* = 8.7, 6.2 Hz, 2H), 7.95 (q, *J* = 9.2 Hz, 2H), 7.87 (d, *J* = 8.2 Hz, 1H), 7.40 (d, *J* = 8.2 Hz, 1H), 4.56 (s, 2H) ppm. $^{13}$C-NMR ($CDCl_3$, 101 MHz, 25 °C) $\delta$ = 141.78, 131.29, 130.75, 130.29, 129.37, 126.88, 125.81, 125.55, 124.12, 122.38, 120.55, 118.89, 116.84, 114.67 ppm.

### Synthesis of 6-bromo-1-dimethylaminopyrene

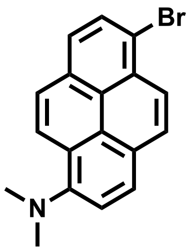


1-Amino-6-bromopyrene (293 mg, 0.991 mmol) and $K_2CO_3$ (702 mg, 5.08 mmol, 5.1 eq.) was dispersed in DMF (11 mL), nitrogen-purged (15 min) and subsequently mixed with iodomethane (350 µL, 798 mg, 5.62 mmol, 5.7 eq.). The reaction mixture was heated to 120 °C and stirred for one hour. Afterwards, it was cooled to RT, quenched with water (20 mL), and the aqueous phase was extracted with DCM (40 mL). Removing the solvent *in vacuo* yielded the product quantitatively. Yield: 321 mg (99.9 %). $^1$H-NMR ($CDCl_3$, 400 MHz, 25 °C) $\delta$ = 8.47 (d, *J* = 9.3 Hz, 1H), 8.27 (d, *J* = 9.2 Hz, 1H), 8.18 (d, *J* = 8.2 Hz, 1H), 8.13 (dd, *J* = 8.3, 1.3 Hz, 1H), 8.06 (dd, *J* = 9.1, 1.3 Hz, 1H), 8.00 (d, *J* = 9.2 Hz, 1H), 7.94 (dd, *J* = 8.2, 1.3 Hz, 1H), 7.76 (dd, *J* = 8.2, 0.9 Hz, 1H), 3.06 (s, 6H) ppm. $^{13}$C-NMR ($CDCl_3$, 101 MHz, 25 °C) $\delta$ = 149.80, 130.93, 130.22, 129.10, 126.81, 126.57, 126.04, 125.52, 124.91, 124.49, 124.08, 123.96, 119.24, 117.18, 45.80 ppm. LC-MS calculated [$C_{18}H_{14}BrN^+$]: 323.0304 g·mol$^{-1}$, found [$C_{18}H_{14}BrN^+$]: 323.0302 g·mol$^{-1}$.

## Synthesis of 1-dimethylamino-6-pentafluorstyryl-pyrene (StyPy)

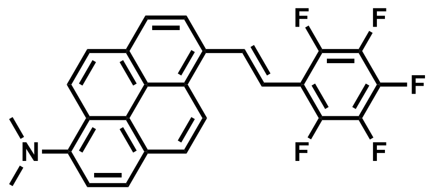


6-Bromo-1-dimethylaminopyrene (321 mg, 0.9898 mmol), $Pd(OAc)_2$ (11.6 mg, 0.0517 mmol, 5.2 mol-%) and $PPh_3$ (61.6 mg, 0.235 mmol, 24 mol-%) were dissolved in DMF (20 mL) and nitrogen-purged (10 min). The solution was mixed with $K_2CO_3$ (144 mg, 1.04 mmol, 1.1 eq.), $NEt_3$ (1.5 mL, 10.8 mmol, 10.9 eq.) and pentafluorostyrene (550 µL, 773 mg, 3.98 mmol, 4.0 eq.). The reaction mixture was heated to 110 °C and stirred for 48 h in a sealed Schlenk flask to the exclusion of light and air. Subsequently, it was mixed with water (40 mL) and the aqueous phase was extracted with chloroform (3 x 100 mL). The combined organic phases were removed *in vacuo* and the raw product was purified *via* flash column chromatography ($SiO_2$) using DCM as the eluent. Remaining impurities were removed by recrystallization from acetone yielding the pure product as a yellow powder. Yield: 270 mg (62.3 %). $^{1}$H-NMR ($CDCl_3$, 400 MHz, 25 °C) $\delta$ = 8.52 (d, $J$ = 16.4 Hz, 2H), 8.27 (t, $J$ = 9.4 Hz, 2H), 8.14 (d, $J$ = 8.2 Hz, 2H), 8.07 (d, $J$ = 9.2 Hz, 2H), 7.77 (d, $J$ = 8.2 Hz, 1H), 7.19 (d, $J$ = 16.5 Hz, 1H), 3.09 (s, 6H) ppm. $^{19}$F-NMR ($CDCl_3$, 377 MHz, 25 °C) $\delta$ = -142.71 (dd, $J$ = 21.7, 7.7 Hz, 2F), -156.55 (1F), -162.83 (td, $J$ = 21.3, 7.6 Hz, 2F) ppm. $^{13}$C-NMR ($CDCl_3$, 101 MHz, 25 °C) $\delta$ = 128.39, 126.05, 116.92, 45.93 ppm. ESI-MS: calculated [$C_{26}H_{17}F_5N^+$]: 438.1276 g·mol$^{-1}$, found [$C_{26}H_{17}F_5N^+$]: 438.1279 g·mol$^{-1}$.

## Functionalization of StyPy

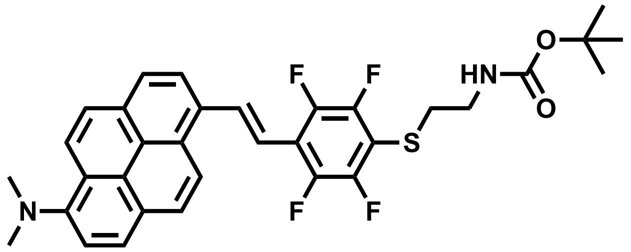


StyPy (20.2 mg, 46.2 µmol) was dispersed in DMF (4 mL) and mixed with DBU (5.9 µL, 14.1 mg, 92.6 µmol, 2.0 eq.) as well as with *Boc*-protected cysteamine. The yellow dispersion turned instantly to a clear orange solution, which was heated to 60 °C and stirred for 2 h. Subsequently, it was mixed with chloroform (20 mL) and washed with water (3 x 15 mL). The solvent was removed *in vacuo* yielding the desired product quantitatively. $^{1}$H-NMR ($CDCl_3$, 400 MHz, 25 °C) $\delta$ = 8.58 (d, $J$ = 16.5 Hz, 1H), 8.48 (d, $J$ = 9.2 Hz, 1H), 8.28 (d, $J$ = 8.2 Hz, 1H), 8.25 (d, $J$ = 9.3 Hz, 1H), 8.13 (dd, $J$ = 8.2, 1.7 Hz, 2H), 8.06 (dd, $J$ = 9.3, 2.3 Hz, 2H), 7.76 (d, $J$ = 8.3 Hz, 1H), 7.25 (m, 1H), 4.99 (s, 1H), 3.33 (q, $J$ = 6.1 Hz, 2H), 3.08 (m, 2H), 1.45 (s, 9H) ppm. $^{19}$F-NMR ($CDCl_3$, 377 MHz, 25 °C) $\delta$ = -134.94 (dd, $J$ = 23.2, 11.4 Hz), -142.24 (m) ppm.

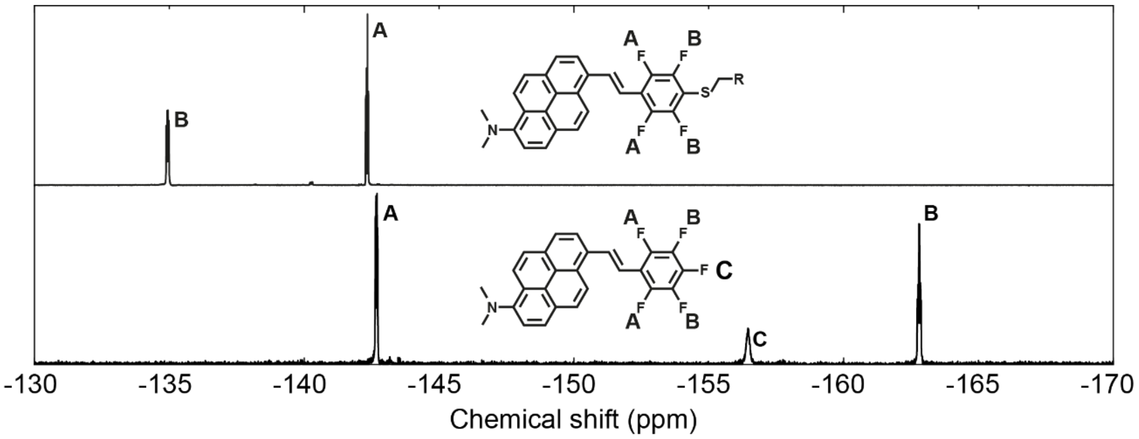


**Figure S1: $^{19}$F-NMR spectra before (bottom) and after (top) PFTR.** Signals are assigned to the fluorine atoms.

## Synthesis of StyPy-k

To a solution of the thiolated ssDNA strand k (140 nmol, 1.13 mg) dissolved in phosphate buffer (10 mM, pH 7.5, 0.5 mL) TCEP (5.0 eq.) was added and stirred for 1 h at 37°C. The resulting reduced thiol-ssDNA was purified by Amicon spin-filtration (3 kDa cut-off, 3 x 10,000 g, 5 min, replaced with MilliQ water).

The reduced ssDNA strand k (0.5 mM, 0.1 eq.) was mixed with StyPy (5 mM) in a mixture of DMSO : MilliQ water (80 : 20 *vol*-%) in presence of DBU (5 mM, 1 eq.). The resulting mixture was reacted at 37 °C for 1 d and DMSO was removed *via* freeze-drying. The resulting precipitate was extracted with MilliQ water and centrifuged to removed excess StyPy (4 x 21,000 g, 20 min). The resulting solution was freeze-dried and the product was obtained as a yellow solid.

## Synthesis of non-fluorescent latex beads

The positively charged latex beads were synthesized by surfactant-free emulsion polymerization of 2,2,2-trifluoroethyl methacrylate (TFEMA), divinylbenzene (DVB) and *N*-isopropylacrylamide (NIPAM) in DI water initiated by 2,2′-azobis(2-methylpropionamidine) dihydrochloride (V50). In detail, TFEMA (2.10 mL, 2.48 g, 14.8 mmol) and DVB (21.0 µL, 19.2 mg, 0.147 mmol) were purified *via* column chromatography ($Al_2O_3$, neutral). The two monomers were mixed with NIPAM (150 mg, 1.32 mmol) dissolved in deionized water (3.9 mL) and the resulting emulsion was degassed by nitrogen (5 min). V50 (160 mg, 0.590 mmol) was dissolved in deionized water (44 mL) and the solution was nitrogen-purged (15 min) while heated to 70 °C. As soon as it reached 70 °C, the monomer-emulsion was added to start the reaction. The reaction mixture was stirred for 3 hours (300 RPM) and the resulting dispersion was filtered while hot and dialyzed against deionized water (5 days, water exchange twice a day) to remove residual reactants. $r_{TEM}$ = 102 ± 4 nm (Figure S8). Zeta potential: $\zeta$ = 6.3 ± 0.7 mV.

## Synthesis of p($A_{20}$-m-XL) and p($T_{20}$-n) DNA polymers

The synthesis of the multiblock DNA polymers were synthesized following our previous report.[1] The 5′-phosphorylated template and its corresponding ligation strand (see Table S2) were mixed (in equimolar amounts) to obtain a final concentration of 1 µM in TE buffer (Invitrogen; 10 mM Tris(hydroxymethyl)aminomethane pH 8 and 1 mM EDTA) containing additionally added 100 mM NaCl (total 100 µL). The buffered DNA mix was heated to 85°C (for 5 min) at a rate of 3°C $s^{-1}$ and cooled to 20°C at 0.01°C $s^{-1}$. After annealing the strands, 20 µL of 10 X commercial ligase buffer (Lucigen; 500 mM TRIS-HCl, 100 mM $MgCl_2$, 50 mM dithiothreitol, and 10 mM ATP), 70 µL of water and 10 µL of $T_4$ Ligase (2 U $\mu{\cdot}L^{-1}$) were mixed to the tube containing 100 µL of the template strand, stirred (10 min, 400 rpm) and left to

react for 4 hours at room temperature. The $T_4$ Ligase was then denatured by heating the reaction mixture for 20 min at 70°C. Then, 10 µL of Exonuclease I (Lucigen; 40 U µ·L$^{-1}$) and 10 µL Exonuclease III (Lucigen; 200 U µ·L$^{-1}$) were added, and the mixture was left overnight at 37°C on a thermo-shaker with gentle stirring (300 rpm) to remove unreacted ligation strands and non-circularized templates in solution. The exonucleases were subsequently deactivated by heating the reaction mixture at 80°C for 40 min. The circular templates were purified by filtration through the Amicon Ultracentrifugal filters with a 10 kDa cut-off (Merck Millipore) and washed 3 times using TE buffer over the same filter. The ssDNA concentrations were measured using a ScanDrop (Jena Analytic) spectrophotometer, and the solutions were diluted to 1 µM using TE buffer. The template synthesis was repeated multiple times, and a stock solution of the circular template was prepared to avoid batch-to-batch discrepancy.

To synthesize multiblock ssDNA polymers via rolling circle amplification (RCA), 10 µL circular template (1 µM) were mixed with 134 µL of ultrapure nuclease-free water, 20 µL of commercial 10 X polymerase buffer (Lucigen; 500 mM TRIS-HCl, 100 mM $(NH_4)_2SO_4$, 40 mM Dithiothreitol, 100 mM $MgCl_2$), 2 µL of exonuclease resistant primer (10 µM in TE buffer), 4 µL of $\Phi_{29}$ Polymerase (Lucigen; 10 U µ·L$^{-1}$), 20 µL of pyrophosphatase (New England Biolabs; 0.1 U µ·L$^{-1}$) and 10 µL of an adjusted dNTP mixture (total dNTP concentration of 100 mM, the percentage of each base corresponds to the expected sequence composition in the ssDNA polymer). The reaction mixtures were kept for 60 hours at 30 °C on a thermo-shaker with gentle stirring (300 rpm). The resulting polymer was then purified by filtration through Amicon Ultra-centrifugal filters with a 30 kDa cut-off (Merck Millipore) and washed thoroughly (3 times) using 400 µL of TE buffer. The concentration of the ssDNAs were determined using a ScanDrop (Jena Analytic) spectrophotometer.

## Synthesis of the raspberry-like DNA-hybrids

A solution of purified p($A_{20}$-m-XL) (0.15 g·L$^{-1}$) in TE buffer was heated up until 95 °C and kept it for 15 min before cooling it down to room temperature. This step is essential to cut the extremely high molecular weight DNA chains and also to homogeneous the solution. The resulting DNA solution was mixed with freshly synthesized non-fluorescent latex particles in an optimized concentration of 0.7 g·L$^{-1}$. A stock solution of $MgAc_2$ (1 M) was added to the mixture to obtain the final concentration of 50 mM. The final colloidal solution was heated to 95 °C for 5 min with heating and cooling ramps of 3 °C·min$^{-1}$. The stoichiometric StyPy-m* was added to hybridize the maximum number of the barcodes present in the hybrids before visualizing them under the CLSM.

## Synthesis of the core-shell DNA microspheres

The core-shell particles were prepared by mixing the starting ssDNA multiblock polymers at the target concentration (for p($A_{20}$-m-XL), typically, around 0.15 g·L$^{-1}$, which corresponds to the barcode-m concentration of 10 µM and for p($T_{20}$-n), around 0.04 g·L$^{-1}$, which corresponds to [barcode-n.] = 2.5 µM in TE buffer (pH 8). Generally, core-shell microspheres were synthesized in 200 µL PCR tubes, and the volume of the PC stock solution varied from 50 µL. The mixture of the two polymers were heated to 95 °C for 15 min for homogenization and thermal cleavage of long ssDNA polymer chains (no phase-separation). A solution of $MgAc_2$ (1 M) was added to the mixture to attain a final concentration of 50 mM (for phase-separation of p($A_{20}$-m-XL)) and subsequently heated to 95 °C for 5 min with heating and cooling ramps of 3 °C·min$^{-1}$. After the formation of the core-shell microspheres, a stoichiometric amount of corresponding fluorescently-labeled barcode* oligomeric sequences (StyPy-m* and $Atto_{647}$-n*) was added to the solution before visualizing them under the CLSM.

## Preparation of the single crystals

StyPy (1.31 mg) was dissolved in chloroform (1.78 mL) to obtain a concentration of $c$ = 1.7 mM. The solution was filtered (0.2 µm pore size, PTFE) and transferred into glass tubes. The glass tubes were placed into a sealed vial, filled with iPrOH (10 mL). The solutions were left in the dark for 10 days, yielding the desired single crystals.

**Table S1: Crystal data and structure refinement for StyPy.**

| | |
|---|---|
| CCDC number | 1986004 |
| Empirical formula | $C_{26}H_{16}F_5N$ |
| Formula weight | 437.40 |
| Temperature (K) | 100(2) |
| Crystal system | monoclinic |
| Space group (number) | $P2_1/c$(14) |
| $a$ (Å) | 17.398(12) |
| $b$ (Å) | 6.826(4) |
| $c$ (Å) | 17.258(11) |
| α (Å) | 90 |
| β (Å) | 111.66(3) |
| γ (Å) | 90 |
| Volume (Å$^3$) | 1905(2) |
| $Z$ | 4 |
| $\rho_{calc}$ (g·cm$^{-3}$) | 1.525 |
| $\mu$ (mm$^{-1}$) | 1.052 |
| $F$(000) | 896 |
| Crystal size (mm$^3$) | 0.300×0.060×0.020 |
| Crystal color | yellow |
| Crystal shape | needle |
| Radiation | Cu$K_\alpha$ ($\lambda$=1.54184 Å) |
| 2Θ range (°) | 5.47 to 150.61 (0.80 Å) |
| Index ranges | -21 ≤ h ≤ 21 |
| | -8 ≤ k ≤ 8 |
| | -21 ≤ l ≤ 21 |
| Reflections collected | 39301 |
| Independent reflections | 3934 |
| | $R_{int}$ = 0.0517 |
| | $R_{sigma}$ = 0.0246 |
| Completeness to θ = 67.684° | 100.0 % |
| Data / Restraints / Parameters | 3934/0/291 |
| Goodness-of-fit on $F^2$ | 1.040 |
| Final $R$ indexes | $R_1$ = 0.0451 |
| [$I \geq 2\sigma(I)$] | w$R_2$ = 0.1251 |
| Final $R$ indexes | $R_1$ = 0.0540 |
| [all data] | w$R_2$ = 0.1332 |
| Largest peak / hole (eÅ$^3$) | 0.45 / -0.21 |

## Quantum yield of fluorescence ($\Phi_{Fl}$)

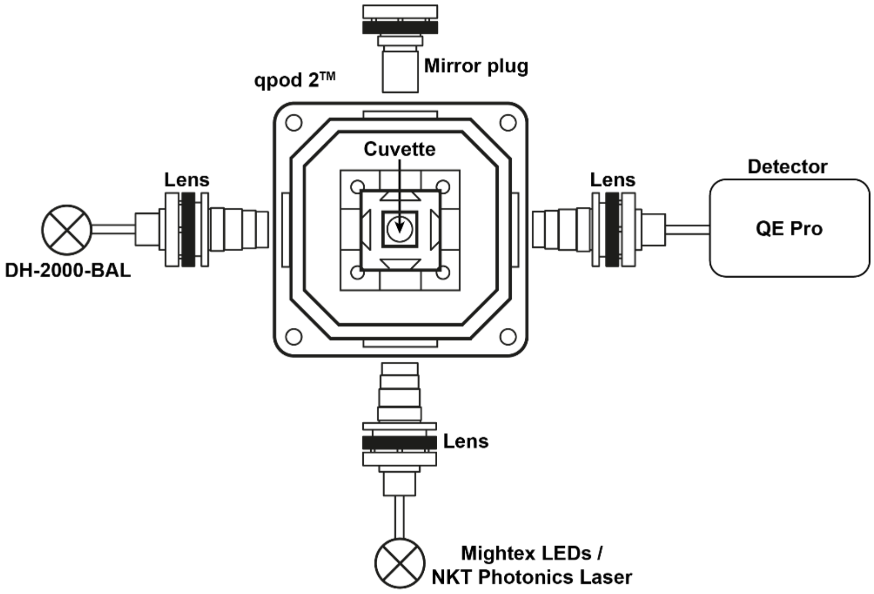


**Figure S2: Experimental set-up to successively measure UV/Vis and fluorescence.**

StyPy and the functionalized StyPy (StyPy-S-Et-NH-*Boc*) were dissolved and diluted in six different solvents to obtain an absorbance $A \leq 0.08$. The samples were irradiated with an EXR-15 supercontinuum white-light laser from NKT Photonics. Monochromatic light of the wavelength of $\lambda_{ex}$ = 425 nm was generated using the SuperK EXTEND-UV filter box from NKT Photonics. The quantum yield of fluorescence was determined in relation to the fluorescent standard BPEA (9,10-bis(phenylethinyl)anthracene) with a quantum yield of $\Phi_R$ = 1.00 in cyclohexane.[2] $\Phi_{Fl}$ was calculated by[3]

$$\Phi_{Fl} = \Phi_R \cdot \frac{Int_X(\mathrm{Fl})}{Int_R(\mathrm{Fl})} \cdot \frac{1-10^{-A_R(425\ \mathrm{nm})}}{1-10^{-A_X(425\ \mathrm{nm})}} \cdot \frac{n_X^2}{n_R^2} \qquad (\mathbf{2})$$

where *Int*(Fl) is the integral of fluorescence, *A*(425 nm) the absorbance at 425 nm, and *n* the refractive index of the solvent, which was obtained from literature.[4] The subscripts R and X denote the reference BPEA and StyPy, respectively. Both the UV/Vis and fluorescence measurements were repeated five times to obtain statistical confidence.

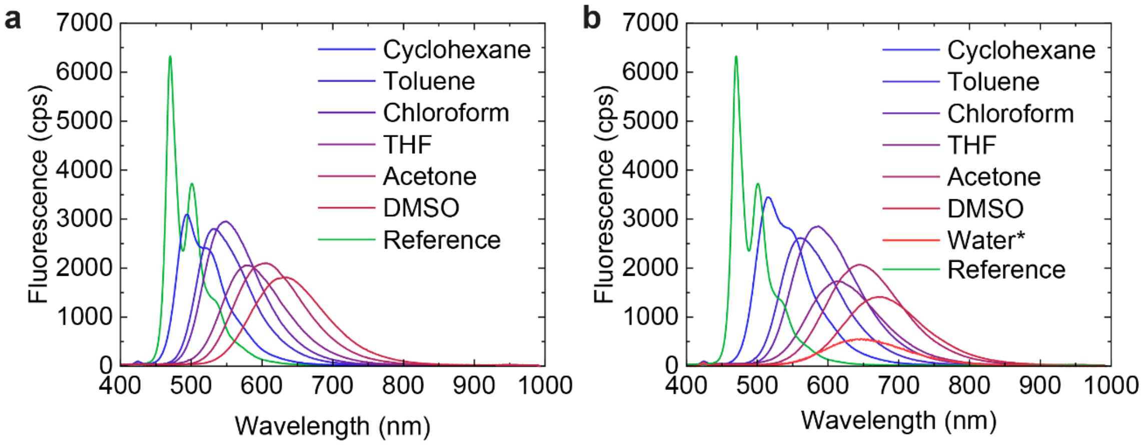


**Figure S3: Quantum yield of fluorescence (a) before and (b) after functionalization.** The fluorescence measurements were recorded in six different solvents and compared to the reference BPEA (green curve). Each measurement was repeated five times to obtain statistical significance ($n$ = 5). The measurement in water, highlighted by a star, was conducted using StyPy-m*.

## Photostability of functionalized StyPy

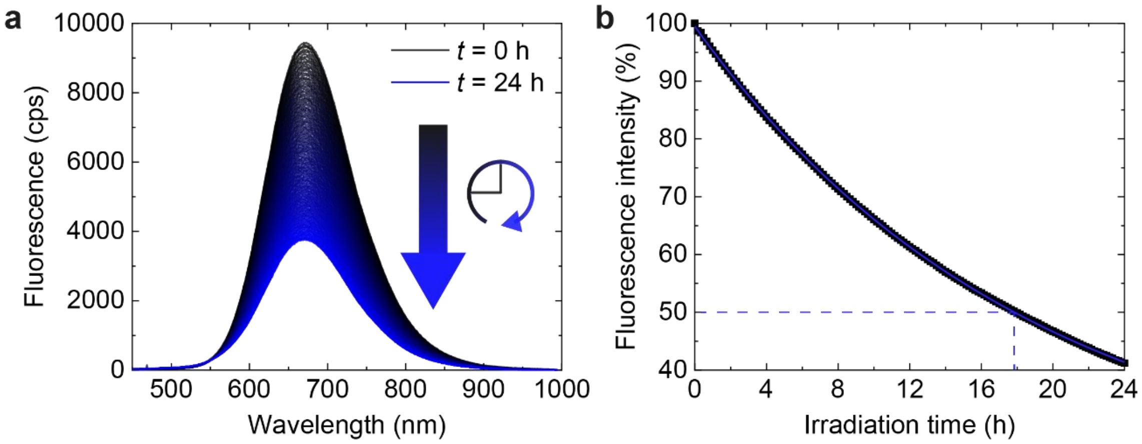


**Figure S4: Photostability of StyPy-S-Et-NH-*Boc* in DMSO (50 µM).** (a) Time-dependent fluorescence spectra recorded over 24 h in intervals of 10 min ($n$ = 145). The sample was continuously irradiated with a high-intensity blue LED (431 ± 7 nm) from Mightex at an intensity of 10 mW·cm$^{-2}$. (b) Fluorescence intensity normalized to the initial intensity as a function of the irradiation time. The half-life of the fluorescence intensity is approximately 18 h, assuming first-order kinetics of photobleaching (dashed lines).

**Table S2: DNA sequences used, with their name, the sequence code used for ordering at IDT**

**The sequence of two final DNA multiblock polymers:**

| | |
|---|---|
| p($A_{20}$-m-XL) | [AAAAAAAAAAAAAAAAAAAA-TTAGGATAGATATACGGGTTC-CGGATGCGCATCCG]$_{15-24}$ |
| p($T_{20}$-n) | [TTTTTTTTTTTTTTTTTTT-GATTTTAGAGGATCGTGTGGTTTTAC]$_{17-31}$ |

| | Name | Sequence 5'→3' | Purification | Modification |
|---|---|---|---|---|
| **Template** | Temp($A_{20}$-m-XL) | /5Phos/ ATC TAT CCT AAT TTT TTT TTT TTT TTT TTT<br>TCG GAT GCG CAT CCG GAA CCC GTA T | HPLC | 5'-Phosphorylation |
| | Temp($T_{20}$-n) | /5Phos/ ATC CTC TAA AAT CAA AAA AAA AAA AAA AAA AAG TAA AAC CAC ACG | HPLC | 5'- Phosphorylation |
| **Ligation** | m | TTA GGA TAG ATA TAC GGG TTC | HPLC | None |
| | n | TTT TAG AGG ATC GTG TGG TTT T | HPLC | None |
| **Primers** | m-exo | TTA GGA TAG ATA TAC GGG T*T*C | Desalting | Phosphorothioated Twice |
| | n-exo | TTT TAG AGG ATC GTG TGG TT*T* T | Desalting | Phosphorothioated Twice |
| **Barcode* strands** | $Atto_{647}$-m* | /5ATTO647NN / TGA ACC CGT ATA TCT ATC CTA A | HPLC | 5' Atto 647N (NHS ester) |
| | $Atto_{488}$-n* | /5ATTO488/ TGA ACC CGT ATA TCT ATC CTA A | HPLC | 5' Atto 488 (NHS ester) |
| | m*-k* | GAACCCGTATATCTATCCTAATTTTCAGTGTCA ATGCGTCGAGGTCTCTG | HPLC | None |
| | Thiol-k | 5'/thiol/-CAGAGACCTCGACGCATTGACACTG | HPLC | 5'-thiolated |

## Characterization of StyPy-ssDNA conjugates

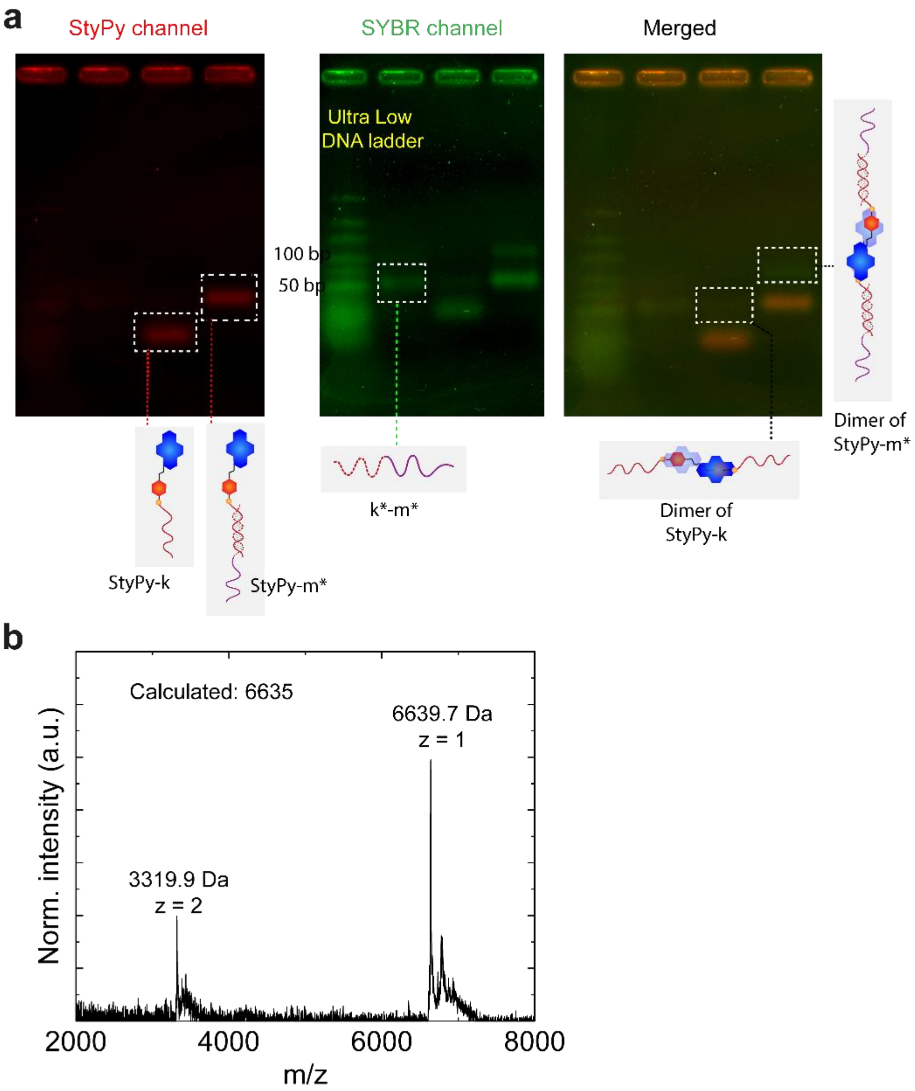


**Figure S5: Characterization of StyPy-DNA conjugates.** (a) Gel-electrophoresis images of the k*-m*, StyPy-k and StyPy-m*. Gel electrophoresis are run in TAE buffer using 3.5 % agarose gel and 6 V/cm for 90 min with SYBR gold staining. A minute amounts of face-to-face StyPy dimers are visible in the SYBR channel. The bands for the ultra-low DNA ladder correspond to 10, 20, 35, 50, 75, 100, 150, 200, 300 base pairs of DNA. (b) MALDI-TOF spectrum of StyPy-k.

## StyPy-labeled DNA microspheres

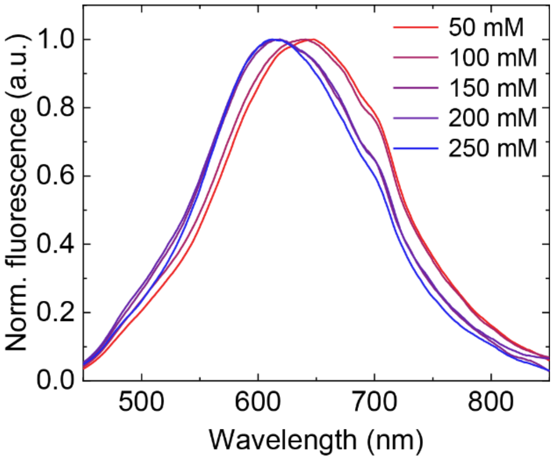


**Figure S6: Mg$^{2+}$-dependent fluorescence spectra of all-DNA microspheres.**

## Control of the function as a viscosity probe

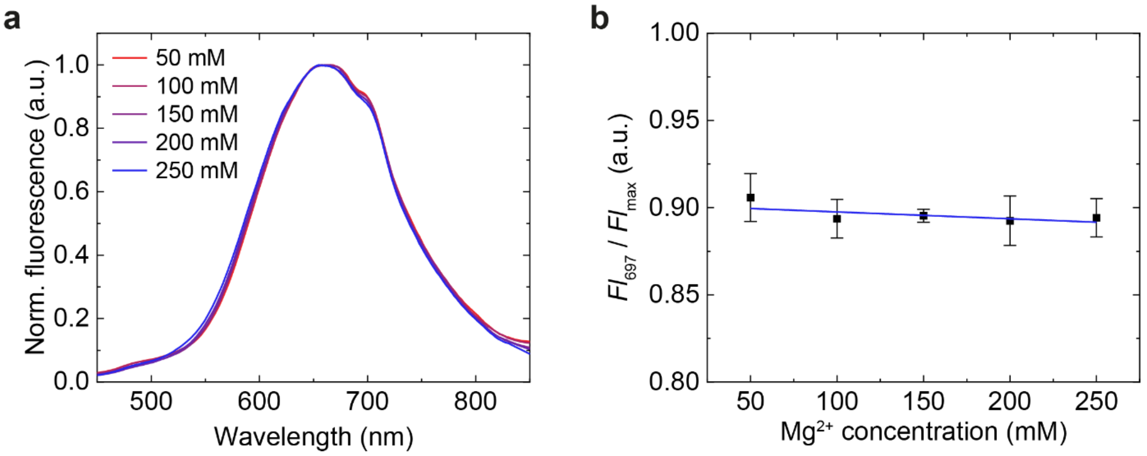


**Figure S7: Control experiments.** (a) Fluorescence spectra of the molecularly dissolved StyPy-DNA hybrid as a function of the Mg$^{2+}$ concentration (exited at 420 ± 2.5 nm). (b) The linear regression reveals that there is a small dependency of the ratio of $FI_{697}$ and $FI_{max}$ on the Mg$^{2+}$ concentration.

## Particle size distribution of non-fluorescent latex beads

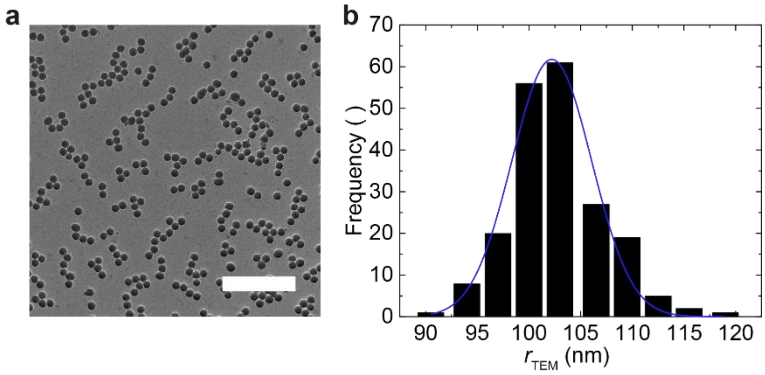


**Figure S8: Characterization of non-fluorescent latex beads.** (a) TEM image of latex beads (scale bar = 2.0 µm). (b) Particle size distribution. The radius of the latex particles was determined *via* TEM image analysis of 200 particles and fitted by a Gaussian curve ($r_{TEM}$ = 102 ± 4 nm).

## Raspberry-like DNA hybrid particles

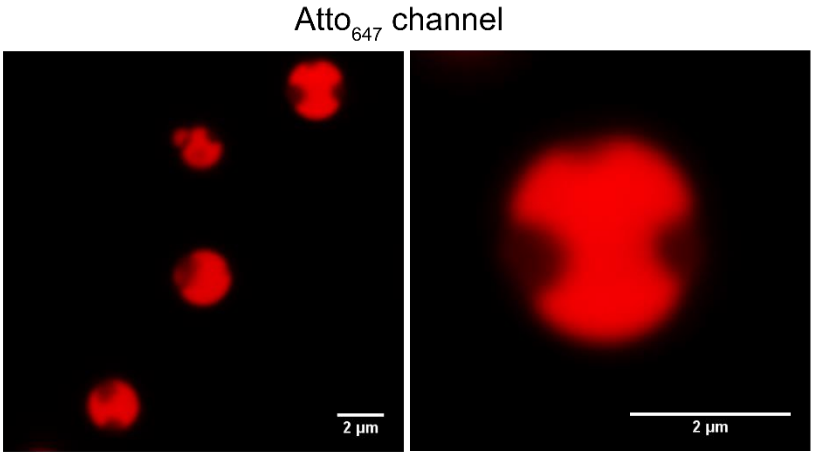


**Figure S9: Atto647-labeled raspberry-like DNA hybrids.**

# References

[1] R. Merindol, S. Loescher, A. Samanta, A. Walther, *Nat. Nanotechnol.* **2018**, *13*, 730-738.

[2] I. Berlman, *Handbook of Fluorescence Spectra of Aromatic Molecules*, Elsevier, **1971**.

[3] A. M. Brouwer, *Pure Appl. Chem.* **2011**, *83*, 2213-2228.

[4] a) I. Z. Kozma, P. Krok, E. Riedle, *J. Opt. Soc. Am. B* **2005**, *22*, 1479-1485; b) Z. S. Baird, P. Uusi-Kyyny, J.-P. Pokki, E. Pedegert, V. Alopaeus, *Int. J. Thermophys.* **2019**, *40*, 102; c) J. Rheims, J. Köser, T. Wriedt, *Meas. Sci. Technol.* **1997**, *8*, 601.

# Appendix

**Table S3: Atomic coordinates and $U_{eq}$ (Å$^2$) of StyPy.**

| Atom | *x* | *y* | *z* | $U_{eq}$ |
|---:|---:|---:|---:|---:|
| C1 | 0.53402(10) | 0.4523(2) | 0.17461(10) | 0.0221(3) |
| H1 | 0.496174 | 0.430945 | 0.119281 | 0.026 |
| F1 | 0.58851(6) | 0.38376(14) | 0.04438(6) | 0.0265(2) |
| C2 | 0.50175(10) | 0.4849(2) | 0.23302(9) | 0.0226(3) |
| H2 | 0.539348 | 0.507730 | 0.288333 | 0.027 |
| F2 | 0.74717(6) | 0.36343(14) | 0.06071(6) | 0.0305(2) |
| C3 | 0.41317(10) | 0.4882(2) | 0.21859(9) | 0.0207(3) |
| F3 | 0.86924(6) | 0.42691(15) | 0.21154(7) | 0.0313(2) |
| C4 | 0.35387(10) | 0.5282(2) | 0.14001(9) | 0.0230(3) |
| H4 | 0.371415 | 0.556558 | 0.095210 | 0.028 |
| F4 | 0.82879(6) | 0.49218(15) | 0.34715(6) | 0.0297(2) |
| C5 | 0.27019(10) | 0.5277(2) | 0.12557(9) | 0.0235(3) |
| H5 | 0.231486 | 0.554257 | 0.071104 | 0.028 |
| F5 | 0.67284(6) | 0.51207(14) | 0.33360(6) | 0.0272(2) |
| C6 | 0.24172(9) | 0.4889(2) | 0.18981(10) | 0.0209(3) |
| C7 | 0.15542(10) | 0.4826(2) | 0.17688(10) | 0.0239(3) |
| H7 | 0.115860 | 0.501704 | 0.122176 | 0.029 |
| C8 | 0.12849(10) | 0.4503(2) | 0.23989(10) | 0.0240(3) |
| H8 | 0.070758 | 0.446735 | 0.228556 | 0.029 |
| C9 | 0.18585(10) | 0.4213(2) | 0.32374(10) | 0.0210(3) |
| C10 | 0.15946(10) | 0.3887(2) | 0.39105(10) | 0.0231(3) |
| C11 | 0.21806(10) | 0.3504(2) | 0.46961(10) | 0.0251(3) |
| H11 | 0.200438 | 0.326502 | 0.514710 | 0.030 |
| C12 | 0.30175(10) | 0.3464(2) | 0.48359(10) | 0.0241(3) |
| H12 | 0.340446 | 0.319040 | 0.537968 | 0.029 |
| C13 | 0.33011(10) | 0.3820(2) | 0.41907(9) | 0.0202(3) |
| C14 | 0.41579(10) | 0.3783(2) | 0.43174(9) | 0.0216(3) |
| H14 | 0.454917 | 0.351455 | 0.485967 | 0.026 |
| C15 | 0.44340(9) | 0.4120(2) | 0.36890(9) | 0.0207(3) |
| H15 | 0.501087 | 0.408610 | 0.379976 | 0.025 |
| C16 | 0.38633(9) | 0.4526(2) | 0.28570(9) | 0.0177(3) |
| C17 | 0.30025(9) | 0.4542(2) | 0.27125(9) | 0.0186(3) |
| C18 | 0.27163(9) | 0.4201(2) | 0.33794(9) | 0.0188(3) |
| C19 | 0.04701(12) | 0.2799(3) | 0.43258(13) | 0.0405(4) |
| H19A | -0.013405 | 0.268388 | 0.410082 | 0.061 |
| H19B | 0.065063 | 0.344495 | 0.487110 | 0.061 |
| H19C | 0.071733 | 0.149064 | 0.438743 | 0.061 |
| C26 | 0.68728(10) | 0.4744(2) | 0.26380(10) | 0.0220(3) |
| C25 | 0.76894(10) | 0.4665(2) | 0.27226(10) | 0.0232(3) |
| C24 | 0.78979(10) | 0.4311(2) | 0.20371(10) | 0.0230(3) |
| C23 | 0.72840(10) | 0.4008(2) | 0.12779(10) | 0.0228(3) |
| C22 | 0.64633(10) | 0.4099(2) | 0.12051(9) | 0.0217(3) |
| C21 | 0.62238(10) | 0.4467(2) | 0.18827(10) | 0.0205(3) |
| C20 | 0.04039(11) | 0.5939(3) | 0.36840(13) | 0.0371(4) |
| H20A | 0.054008 | 0.665291 | 0.325889 | 0.056 |
| H20B | 0.064893 | 0.661274 | 0.422090 | 0.056 |
| H20C | -0.019819 | 0.588531 | 0.352205 | 0.056 |
| N1 | 0.07319(9) | 0.3955(2) | 0.37585(9) | 0.0277(3) |

$U_{eq}$ is defined as 1/3 of the trace of the orthogonalized $U_{ij}$ tensor.

**Table S4: Bond lengths of StyPy.**

| Atom-Atom | Length (Å) |
|---|---|
| C1–C21 | 1.467(2) |
| C1–H1 | 0.9500 |
| F1–C22 | 1.340(2) |
| C2–C3 | 1.468(2) |
| C2–H2 | 0.9500 |
| F2–C23 | 1.3383(18) |
| C3–C4 | 1.395(2) |
| C3–C16 | 1.421(2) |
| F3–C24 | 1.339(2) |
| C4–C5 | 1.383(2) |
| C4–H4 | 0.9500 |
| F4–C25 | 1.339(2) |
| C5–C6 | 1.396(2) |
| C5–H5 | 0.9500 |
| F5–C26 | 1.3431(19) |
| C6–C17 | 1.418(2) |
| C6–C7 | 1.435(2) |
| C7–C8 | 1.352(2) |
| C7–H7 | 0.9500 |
| C8–C9 | 1.436(2) |
| C8–H8 | 0.9500 |
| C9–C10 | 1.415(2) |
| C9–C18 | 1.420(2) |
| C10–C11 | 1.388(2) |
| C10–N1 | 1.426(2) |
| C11–C12 | 1.385(2) |
| C11–H11 | 0.9500 |
| C12–C13 | 1.396(2) |
| C12–H12 | 0.9500 |
| C13–C18 | 1.418(2) |
| C13–C14 | 1.425(2) |
| C14–C15 | 1.358(2) |
| C14–H14 | 0.9500 |
| C15–C16 | 1.440(2) |
| C15–H15 | 0.9500 |
| C16–C17 | 1.424(2) |
| C17–C18 | 1.432(2) |
| C19–N1 | 1.455(2) |
| C19–H19A | 0.9800 |
| C19–H19B | 0.9800 |
| C19–H19C | 0.9800 |
| C26–C25 | 1.375(2) |
| C26–C21 | 1.387(2) |
| C25–C24 | 1.381(2) |
| C24–C23 | 1.367(3) |
| C23–C22 | 1.388(2) |
| C22–C21 | 1.402(2) |
| C20–N1 | 1.456(2) |
| C20–H20A | 0.9800 |
| C20–H20B | 0.9800 |
| C20–H20C | 0.9800 |

**Table S5: Dihedral angles of StyPy.**

| Atom-Atom-Atom | Angle (°) |
|---|---|
| C2–C1–C21 | 126.02(15) |
| C2–C1–H1 | 117.0 |
| C21–C1–H1 | 117.0 |
| C1–C2–C3 | 125.42(15) |
| C1–C2–H2 | 117.3 |
| C3–C2–H2 | 117.3 |
| C4–C3–C16 | 118.59(15) |
| C4–C3–C2 | 121.43(14) |
| C16–C3–C2 | 119.97(14) |
| C5–C4–C3 | 121.75(15) |
| C5–C4–H4 | 119.1 |
| C3–C4–H4 | 119.1 |
| C4–C5–C6 | 121.03(15) |
| C4–C5–H5 | 119.5 |
| C6–C5–H5 | 119.5 |
| C5–C6–C17 | 118.89(15) |
| C5–C6–C7 | 122.67(15) |
| C17–C6–C7 | 118.44(14) |
| C8–C7–C6 | 122.24(15) |
| C8–C7–H7 | 118.9 |
| C6–C7–H7 | 118.9 |
| C7–C8–C9 | 120.98(15) |
| C7–C8–H8 | 119.5 |
| C9–C8–H8 | 119.5 |
| C10–C9–C18 | 119.73(14) |
| C10–C9–C8 | 122.23(15) |
| C18–C9–C8 | 118.01(14) |
| C11–C10–C9 | 119.19(15) |
| C11–C10–N1 | 121.98(15) |
| C9–C10–N1 | 118.83(15) |
| C12–C11–C10 | 121.31(15) |
| C12–C11–H11 | 119.3 |
| C10–C11–H11 | 119.3 |
| C11–C12–C13 | 121.06(15) |
| C11–C12–H12 | 119.5 |
| C13–C12–H12 | 119.5 |
| C12–C13–C18 | 118.92(15) |
| C12–C13–C14 | 122.17(15) |
| C18–C13–C14 | 118.91(14) |
| C15–C14–C13 | 122.20(15) |
| C15–C14–H14 | 118.9 |
| C13–C14–H14 | 118.9 |
| C14–C15–C16 | 120.80(15) |
| C14–C15–H15 | 119.6 |
| C16–C15–H15 | 119.6 |
| C3–C16–C17 | 119.78(14) |
| C3–C16–C15 | 122.20(14) |
| C17–C16–C15 | 118.01(14) |
| C6–C17–C16 | 119.90(14) |
| C6–C17–C18 | 119.23(15) |
| C16–C17–C18 | 120.87(14) |
| C13–C18–C9 | 119.77(14) |
| C13–C18–C17 | 119.20(15) |
| C9–C18–C17 | 121.03(14) |
| N1–C19–H19A | 109.5 |
| N1–C19–H19B | 109.5 |
| H19A–C19–H19B | 109.5 |
| N1–C19–H19C | 109.5 |
| H19A–C19–H19C | 109.5 |
| H19B–C19–H19C | 109.5 |
| F5–C26–C25 | 116.12(14) |

| | |
|---|---|
| F5–C26–C21 | 120.82(14) |
| C25–C26–C21 | 123.06(15) |
| F4–C25–C26 | 120.17(15) |
| F4–C25–C24 | 119.57(15) |
| C26–C25–C24 | 120.26(15) |
| F3–C24–C23 | 120.40(15) |
| F3–C24–C25 | 120.36(15) |
| C23–C24–C25 | 119.24(15) |
| F2–C23–C24 | 120.29(15) |
| F2–C23–C22 | 120.11(15) |
| C24–C23–C22 | 119.60(15) |
| F1–C22–C23 | 117.26(14) |
| F1–C22–C21 | 119.69(15) |
| C23–C22–C21 | 123.04(15) |
| C26–C21–C22 | 114.78(15) |
| C26–C21–C1 | 126.07(14) |
| C22–C21–C1 | 119.16(14) |
| N1–C20–H20A | 109.5 |
| N1–C20–H20B | 109.5 |
| H20A–C20–H20B | 109.5 |
| N1–C20–H20C | 109.5 |
| H20A–C20–H20C | 109.5 |
| H20B–C20–H20C | 109.5 |
| C10–N1–C19 | 115.35(14) |
| C10–N1–C20 | 113.47(13) |
| C19–N1–C20 | 110.71(15) |
